# LnCeVar 2.0: an updated resource and web tools for genomic variations disrupting ceRNA networks from single-cell/spatial transcriptomics data

**DOI:** 10.1093/nar/gkaf1009

**Published:** 2025-10-16

**Authors:** Qiuyan Guo, Qian Liu, Mengyu Xin, Houxing Li, Jiatong Li, Yifan Dai, Rui Sun, Yujie Zhang, Yuting He, Borui Xu, Xinxin Shan, Yue Gao, Shangwei Ning, Hui Zhi, Peng Wang

**Affiliations:** Department of Gynecology, the First Affiliated Hospital of Harbin Medical University, Harbin 150081, China; College of Bioinformatics Science and Technology, Harbin Medical University, Harbin 150081, China; College of Bioinformatics Science and Technology, Harbin Medical University, Harbin 150081, China; College of Bioinformatics Science and Technology, Harbin Medical University, Harbin 150081, China; College of Bioinformatics Science and Technology, Harbin Medical University, Harbin 150081, China; College of Bioinformatics Science and Technology, Harbin Medical University, Harbin 150081, China; College of Bioinformatics Science and Technology, Harbin Medical University, Harbin 150081, China; College of Bioinformatics Science and Technology, Harbin Medical University, Harbin 150081, China; College of Bioinformatics Science and Technology, Harbin Medical University, Harbin 150081, China; College of Bioinformatics Science and Technology, Harbin Medical University, Harbin 150081, China; College of Bioinformatics Science and Technology, Harbin Medical University, Harbin 150081, China; College of Bioinformatics Science and Technology, Harbin Medical University, Harbin 150081, China; College of Bioinformatics Science and Technology, Harbin Medical University, Harbin 150081, China; College of Bioinformatics Science and Technology, Harbin Medical University, Harbin 150081, China; College of Bioinformatics Science and Technology, Harbin Medical University, Harbin 150081, China

## Abstract

LnCeVar 2.0 (available at http://bio-bigdata.hrbmu.edu.cn/LnCeVar or http://www.bio-bigdata.net/LnCeVar) is an updated database investigating genomic variations that disrupt competing endogenous RNA (ceRNA) networks via single-cell and spatial transcriptomics. Enhancements include expanded data and improved features: (i) 16 937 experimentally supported cancer biomarkers as well as 5785 validated ceRNA interactions and single nucleotide variant (SNV)–ceRNA events, manually curated and linked to key cancer pathogenic processes; (ii) 812 single-cell RNA sequencing/spatial transcriptomics RNA sequencing datasets covering 102 diseases, clinical treatments (e.g. chemotherapy, immunotherapy), and normal tissues; (iii) 5 218 062 single-cell- and spatial-specific SNV–ceRNA events across 2 673 603 cells/spots, with cellular functional perturbation networks; (iv) 5 comprehensive and 12 mini tools for multilevel cross talk analysis and 3D visualization; and (v) novel inference of SNV effects on cell types, states, and functions at single-cell and spatial levels. LnCeVar 2.0 features a user-friendly interface for searching, browsing, and analyzing data. For instance, the *CeVarState* interface illustrates how SNV–ceRNA events influence cell states during developmental processes, revealing interactions that determine cell fate. The *CeVarSC3D* and *CeVarST3D* tools perform multilevel cross talk analyses of SNVs, ceRNA networks, and cell states in disease pathology, providing interactive 3D visualizations. Overall, we anticipate that the updated database will facilitate the high-resolution investigation of SNV–ceRNA networks and advance our understanding of the regulatory mechanisms in complex disease ecosystems.

## Graphical Abstract

**Graphical Abstract:**
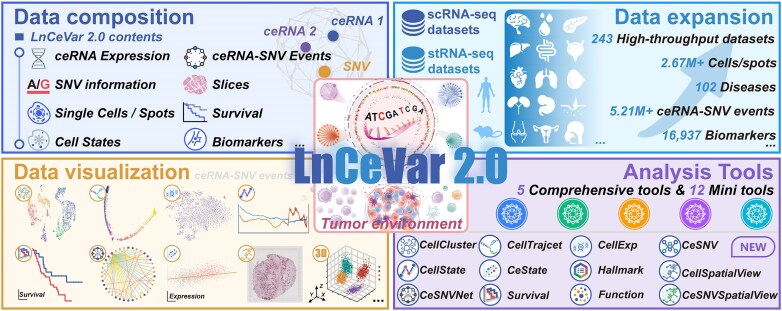

## Introduction

Single nucleotide variants (SNVs) exert diverse functional effects across genome regions, contributing to genetic diversity and individual phenotypic variation [1]. Those occurring in coding regions can alter protein structure, while variants in regulatory regions may disrupt gene regulation by impairing transcription factor–DNA binding or protein–RNA interactions [2, 3]. Notably, SNVs are far more prevalent in non-coding regions, particularly within microRNA (miRNA) binding sites on long non-coding RNAs (lncRNAs) [4]. Such variants can perturb lncRNA–miRNA interactions, thereby disrupting competing endogenous RNA (ceRNA) regulatory networks and influencing disease initiation and progression [5, 6]. For instance, a functional genetic variant, rs67311347 (G > A), increases the expression of ENTPD3-AS1 by forming a ZNF8 binding motif, and this upregulation inhibits renal cell carcinoma via the miR-155/HIF-1α signaling pathway [7]. The lncRNA LOC146880, harboring the single nucleotide polymorphism (SNP) rs140618127, provides a binding site for miR-539-5p, which inhibits ENO1 phosphorylation and downregulates the phosphatidylinositol-3-kinase (PI3K)/Akt pathway, thereby reducing the risk of non-small cell lung cancer [8]. A genome-wide study of 2130 ovarian cancer patients identified a locus at 3p26.1 associated with overall survival and a likely causal variant that alters enhancer activity by interacting with lncRNA BHLHE40-AS1, influencing oncogenic expression and tumor growth [9]. The identification of such personalized variation events will enhance our understanding of individual disease pathology and further contribute to the advancement of precision medicine.

Our previous work, LnCeVar 1.0, curated SNV–ceRNA events from thousands of samples and cell lines, serving as a vital resource for investigating the functional impact of genome variations on ceRNA network regulation in human diseases [10]. Notably, single-cell RNA sequencing (scRNA-seq) and spatial transcriptomics RNA sequencing (stRNA-seq) technologies enable the characterization of cellular heterogeneity and dynamic states within the tumor microenvironment, facilitating the dissection of intercellular interactions and signaling pathways [11]. The stRNA-seq further captures cellular spatial locations within tissues, enabling precise analysis of cell distribution and interactions in the tumor microenvironment [12]. SNVs in the cellular genome disrupt gene regulatory networks, driving changes in cellular states and behaviors, such as metastasis, invasion, and immune escape [13]. Thus, identifying SNVs at the single-cell and spatial levels and further analyzing their impact on gene regulatory networks is crucial for understanding the determinants of cell fate and revealing dynamic changes.

Existing research has developed methods such as RESA [14], scSNV [15], and SComatic [16], which enable the identification of SNVs at single-cell and spatial resolutions. Meanwhile, large-scale SNV resources at the single-cell and spatial resolutions are rapidly emerging, such as CanCellVar [17] and stSNV [18]. To better understand the impact of SNVs within cells on ceRNA regulatory networks and further explore the driving factors behind different cell fates, we developed LnCeVar 2.0. This updated database will enable us to study complicated SNV–ceRNA networks at a single-cell and spatial resolution, helping us to gain a deeper understanding of disease pathology.

LnCeVar 2.0 is an enhanced resource for investigating SNV–ceRNA regulation at cellular/spatial resolutions. It integrates high-throughput scRNA-seq, stRNA-seq, and bulk-seq data from over 20 000 human/mouse normal/diseased samples, identifying 5 218 062 functional SNV–ceRNA events across 2 673 603 cells/spots. It also includes 5785 experimentally validated ceRNA interactions/SNV–ceRNA events and 16 937 manually curated cancer biomarker annotations (linked to pathology, diagnosis, and treatment). With a user-friendly web interface for searching, browsing, downloading, and analyzing, as well as tools for gene expression/SNV analysis, 3D cross talk studies, and more. This update will further enhance our understanding of individual disease pathology and drive advancements in precision medicine.

## Materials and methods

### Data collection and processing

High-throughput scRNA-seq and stRNA-seq datasets were compiled through manual curation of PubMed publications and retrieval from public repositories, including NCBI-GEO [19], 10x Genomics (https://www.10xgenomics.com/), and relevant databases [20–25]. Based on GENCODE annotations (version GRCh38/GRCm39), LnCeVar 2.0 systematically identifies human and mouse gene expression profiles across diverse gene types. Data preprocessing and normalization were performed using the R package Seurat (v4.3) [26], with the FindClusters, RunTSNE, and RunUMAP functions utilized for cell clustering and visualization. The R packages Monocle 2 (v2.18.0) [27] and Monocle 3 (v1.2.9) [28] were employed to calculate pseudotime, construct cell developmental trajectories, and determine cell states. The scSNV [15] and SComatic [16] methods were used to identify SNVs at the single-cell and spatial levels. A multivariate multiple regression (MMR) model was applied to examine the effects of SNVs on ceRNA expression [5, 10, 29]. The impact of functional SNV–ceRNA events was estimated using the corresponding coefficients (*coef-*values) for the SNVs, and the significance (*P-*values) of the MMR model was assessed via Pillai’s trace statistics. All statistical analyses were conducted using the R programming language (v4.2.3). Further details on data collection and processing can be found in the Supplementary Methods.

### Database construction and interface organization

The LnCeVar 2.0 platform was built using MySQL (version 5.5) for data management, with web interfaces developed through Java Server Pages and deployed on the Tomcat web server (v6). Several JavaScript libraries were integrated to enable interactive data visualization and table rendering, including jQuery (v1.11.3), Datatables (v1.10.10), and ECharts (v4.0). The LnCeVar 2.0 database can be accessed at http://bio-bigdata.hrbmu.edu.cn/LnCeVar or http://www.bio-bigdata.net/LnCeVar. The previous version (LnCeVar 1.0, available at http://www.bio-bigdata.net/LnCeVar1.0/) remains accessible for users requiring legacy data or features.

## Results

### New collection of high-throughput scRNA-seq and stRNA-seq data

**Figure 1.**
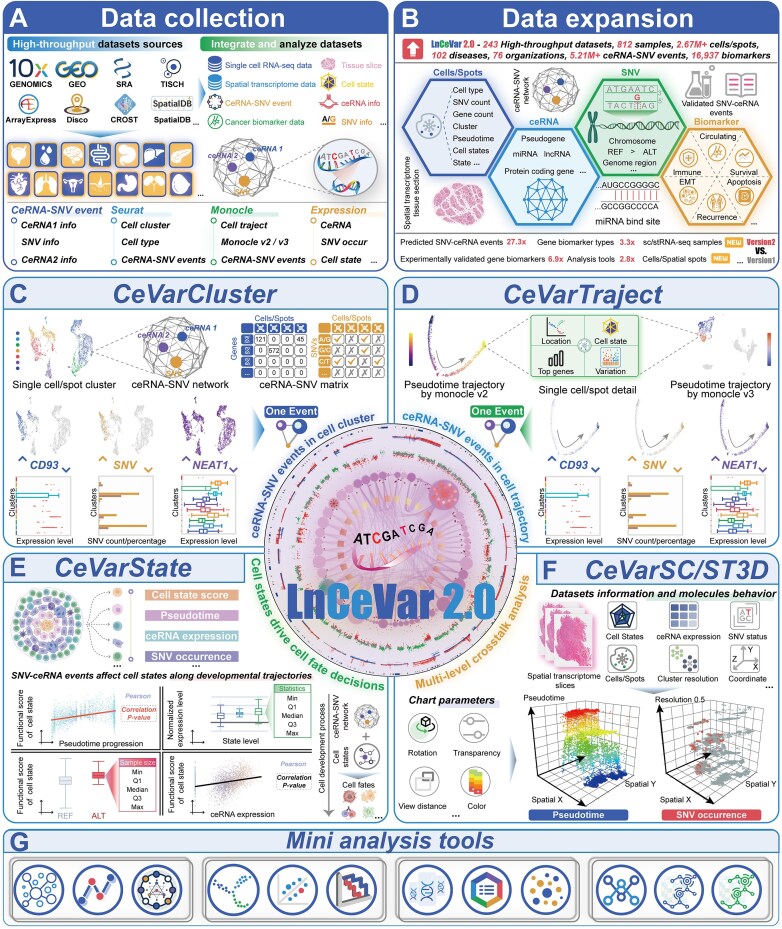
Data expansion and new features of LnCeVar 2.0. (**A**, **B**) Collection and expansion of high-throughput scRNA-seq, stRNA-seq, and bulk-seq datasets of human and mouse. (**C**, **D**) The multidimensional mapping of cellular clusters, developmental trajectories, and spatial positions. (**E**) Functional state profiling, pathway activity quantification, and cancer hallmark enrichment scoring for individual cells/spots. (**F**) Visualizing gene expression in single-cell and spatially resolved tissue architecture and pathology.

To enhance the comprehensiveness and functionality of the LnCeVar 2.0 database, substantial efforts have been devoted to expanding its data content, specifically enriching the high-throughput scRNA-seq and stRNA-seq datasets (Fig. 1A–G). The database now encompasses gene expression profiles of 812 human and mouse samples, collectively covering 1 773 362 cells and 900 241 spatial spots. Following meticulous data collection and preprocessing procedures (Supplementary Methods), 400 distinct cell types were identified, substantially expanding the range of cell types represented in the database. To broaden the disease spectrum, 102 diseases and disease-related phenotypes with diverse clinical trials and outcomes (such as chemotherapy, immunotherapy, targeted gene therapy, and CRISPR gene knockout) have been incorporated. To conduct comparative analyses of the tumor microenvironment at single-cell and spatial levels, we compiled 244 gene expression profiles from normal human/mouse organs and tissues. The aim was to define the cellular composition of major healthy organs and create a landscape of human/mouse cells. Using GENCODE gene type and transcript annotations (version GRCh38/GRCm39), LnCeVar 2.0 systematically identifies human and mouse gene expression profiles across various gene types, including protein-coding genes, lncRNAs, pseudogenes, and other genes, resulting in the identification of over 20 000 coding and noncoding genes from the scRNA-seq and stRNA-seq data.

To fully leverage the high-resolution capabilities of single-cell and spatial omics technologies for advancing gene expression regulation studies in the tumor microenvironment, we have systematically curated and annotated the collected datasets. Each dataset is characterized by the following features: (i) gene expression profiles at single-cell and spatial-spot resolutions (Fig. 1A and B); (ii) multidimensional coordinates mapping cellular clusters, developmental trajectories, and spatial positions (Fig. 1C and D); (iii) functional state profiling, pathway activity quantification, and cancer hallmark enrichment scoring for individual cells and spatial spots (Fig. 1E); and (iv) histological images that enable visualization of gene expression within the context of spatially resolved tissue architecture and disease pathology (Fig. 1F). A panel of tools, by integrating high-resolution single-cell and spatial transcriptomics data, enhances the interpretability of histopathology and facilitates investigating mechanisms that disrupt gene networks (Fig. 1G). This represents a significant expansion compared to LnCeVar 1.0 in terms of dataset quantity, cell numbers, cell types, gene counts, and single-cell and spatial features (Table 1).

**Table 1.** Data expansion and functional enhancements in LnCeVar 2.0

Datasets and features		LnCeVar 1.0	LnCeVar 2.0	Fold increase↑
High-throughput datasets	scRNA-seq samples	–	357	New
	stRNA-seq samples	–	455	New
	Bulk-seq samples	11 432	22 515	1.97↑
	Single cells	–	1 773 362	New
	Cell types	–	400	New
	Spatial cells/spots	–	900 241	New
	Spatial slices	–	478	New
	Diseases/phenotypes	58	102	1.76↑
	Normal tissues/organs	28	80	2.86↑
	Species	Human	Human, mouse	New
Genetic variations, gene annotations, and regulatory events	Variations	191 158	2 547 153	13.32↑
	Coding genes	–	21 639	New
	Noncoding genes	1845	3272	1.77↑
	Predicted SNV–ceRNA events	191 158	5 218 062	27.30↑
	Validated SNV–ceRNA events and ceRNA interactions	54	5785	107.13↑
	Experimentally validated diagnostics and therapy biomarkers	2444	16 937	6.93↑
	Category of diagnostics and therapy biomarkers	3	10	3.33↑
Function features and analysis tools	Functional gene sets	7246	32 139	4.44↑
	Mini analysis tools	6	12	2.00↑
	Comprehensive analysis tools	–	5	New
	Cell cluster analysis	–	√	New
	Cell trajectory analysis	–	√	New
	Cell type annotation	–	√	New
	Cell state annotation	–	√	New
	scRNA-seq data analysis	–	√	New
	stRNA-seq data analysis	–	√	New
	Multilevel correlation analysis	–	√	New
	3D-data visualization	–	√	New

### Newly identified functional single-cell and spatially specific SNV–ceRNA events

LnCeVar 2.0 provides a comprehensive profile of genomic SNVs in human and mouse, revealing the co-localization of SNVs in distinct cell clusters along with their unique characteristics and enabling exploration of the profound impact of SNVs on gene expression at the single-cell and spatial levels (Supplementary Fig. S1). Following the collection and processing of SNV data, a total of 1 839 720 and 707 433 SNVs were identified in over 70 human and mouse tissues, respectively. LnCeVar 2.0 compiles ceRNA candidates from existing databases [30–34], providing information on potential single-cell and spatially specific ceRNA interactions. A published method based on probability theory was applied to construct cell-specific networks and identify ceRNAs at single-cell and spatial resolutions [35]. A candidate SNV–ceRNA event was identified if different genotypes of a variation could alter ceRNA regulations (e.g. gain, loss, or alternation of miRNA binding sites). To further identify functional SNV–ceRNA events at the expression level, the MMR model was employed to examine whether a given SNV regulates the expression of both ceRNA genes [5, 10, 29]. The effect of functional SNV–ceRNA events was estimated using the corresponding coefficients for the SNV (details in Supplementary Methods and Supplementary Fig. S2). Ultimately, a total of 5 218 062 functional SNV–ceRNA events were identified across 2 673 603 single cells and spatial spots. Collectively, these functional single-cell and spatially specific SNV–ceRNA events will facilitate investigations into fine-tuned SNV–ceRNA networks at single-cell and spatial resolutions, aiding to the understanding of regulatory mechanisms underlying complex biological ecosystems.

### Curation of validated SNV–ceRNA events, biomarkers, and functional annotations

To provide more detailed and reliable information regarding the mechanisms of SNV and ceRNA regulation, LnCeVar 2.0 has been updated to include an expanded set of experimentally verified ceRNA interactions and SNV–ceRNA events, which were collated from our previous studies [30–34, 36] and manual curation (Supplementary Methods). Specifically, a total of 5785 validated ceRNA interactions and SNV–ceRNA events were confirmed using high-confidence experimental techniques, including polymerase chain reaction, western blotting, luciferase reporter assays, and other methodologies. Additionally, LnCeVar 2.0 conducted manual curation of ceRNA and SNV annotations related to cancer biomarkers associated with disease pathology, diagnosis, and treatment. Following manual curation, a total of 16 937 experimentally supported biomarkers were incorporated into LnCeVar 2.0. Furthermore, LnCeVar 2.0 contains 18 100 and 14 039 functional gene sets for the human and mouse genomes, respectively. These gene sets encompass a wide range of functional annotations, including Gene Ontology [37], canonical pathways [38], cancer cell states [39], and classical cancer hallmarks [40], thereby facilitating comprehensive functional analysis (Supplementary Fig. S3). To infer the regulatory impact of SNV–ceRNA events on clinical characteristics, LnCeVar 2.0 integrated bulk-seq data and clinical profiles from 22 515 tumor samples across various types. These datasets, sourced from TCGA [41] and NCBI-GEO [19], encompass ceRNA expression, genome variation across individuals, clinicopathological features, clinical treatment details, and follow-up data. The incorporation of cancer biomarkers, functional annotations, and clinical features into LnCeVar 2.0 provides a more robust framework for investigating ceRNA regulatory mechanisms through pathological analysis.

### Novel data features and enhanced analysis tools

Recent advances in single-cell and spatial transcriptomics have revolutionized the dissection of molecular heterogeneity in disease, enabling unprecedented resolution to explore how genomic variations disturb gene expression, regulatory networks, and cellular phenotypes in the tumor microenvironment. To better leverage such data, LnCeVar 2.0 has been updated with several new features for exploring the regulatory roles of SNV–ceRNA events at the single-cell and spatial levels, thereby facilitating the study of fine-tuned SNV–ceRNA effects to unravel the regulatory mechanisms underlying complex ecosystems. A range of flexible tools, including 5 comprehensive and 12 mini analysis tools, have been developed to facilitate data retrieval and analysis (Supplementary Figs S4 and S5). For example, the *CeVarCluster* tool examines the patterns of gene expression and SNV mutations in SNV–ceRNA events while analyzing the distribution of cell clusters, types, states, and trajectories. The *CeVarTraject* tool illustrates the dynamic changes in SNV genotype and gene expression variation along cell differentiation trajectories. The *CeVarState* tool evaluates the driver effect of SNV–ceRNA events on cell states along developmental trajectories, revealing the dynamic interactions that determine cell fate. The *CeVarSC3D* and *CeVarST3D* tools enable multilevel cross talk analyses of SNVs, ceRNA networks, and cell states contributing to disease pathology at single-cell and spatial levels, with results presented via an interactive 3D view. Each of the 12 mini tools provides fast, user-friendly analysis including functional, hallmark and cell state annotations, cell clustering, survival and correlation analyses, and SNV–ceRNA network construction.

### A usage example of LnCeVar 2.0 database

**Figure 2.**
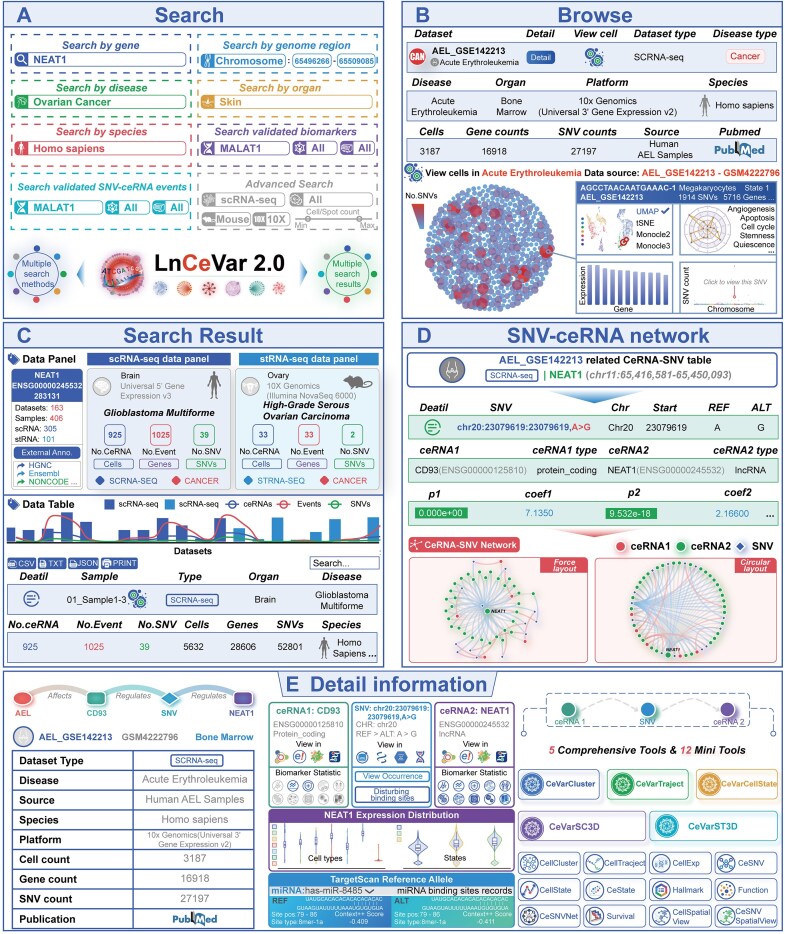
The data query and analysis workflow of LnCeVar 2.0 database. (**A**, **B**) Searching and browsing interfaces of LnCeVar 2.0 database with advanced options. (**C**) Search results show *NEAT1*-related basic gene info, associated counts of ceRNAs, SNV–ceRNA events, and related disease/species. (**D**, **E**) Detail page with disease–SNV–ceRNA associations, showing how SNV genotypes affect ceRNA regulation and downstream gene expression across single-cell/spatial omics in diverse diseases.

LnCeVar 2.0 provides a convenient, user-friendly web interface for data searching, browsing, analysis, and downloading (Fig. 2A–E). Its “HOME” page includes search and browse interfaces (Supplementary Figs S6 and S7) with keywords such as different gene types (lncRNAs, mRNAs, pseudogenes), diseases, organs, and SNV genomic regions (Fig. 2A and B). To illustrate the database’s functionality, a case study on *NEAT1*-related data is presented through interactive panels and tables, enabling users to filter and reorder results via table headers (Fig. 2A). All *NEAT1*-related basic gene information (including gene name, annotated gene IDs from external databases, etc.), as well as the number of associated ceRNAs, SNV–ceRNA events, related disease information, and species information, are displayed in the search results (Fig. 2C and Supplementary Figs S8 and S9). The “Detail” column directs users to a dedicated page containing comprehensive information on disease–SNV–ceRNA associations, showcasing how different SNV genotypes affect ceRNA regulatory status and further impact downstream gene expression across single-cell and spatial omics data from various diseases (Fig. 2D and E). Additionally, information is provided on whether *NEAT1* and its downstream targets have been validated in experiments as cancer biomarkers associated with cancer cell growth, metastasis, disease recurrence, survival, drug resistance, and immune escape (Supplementary Fig. S10).

**Figure 3.**
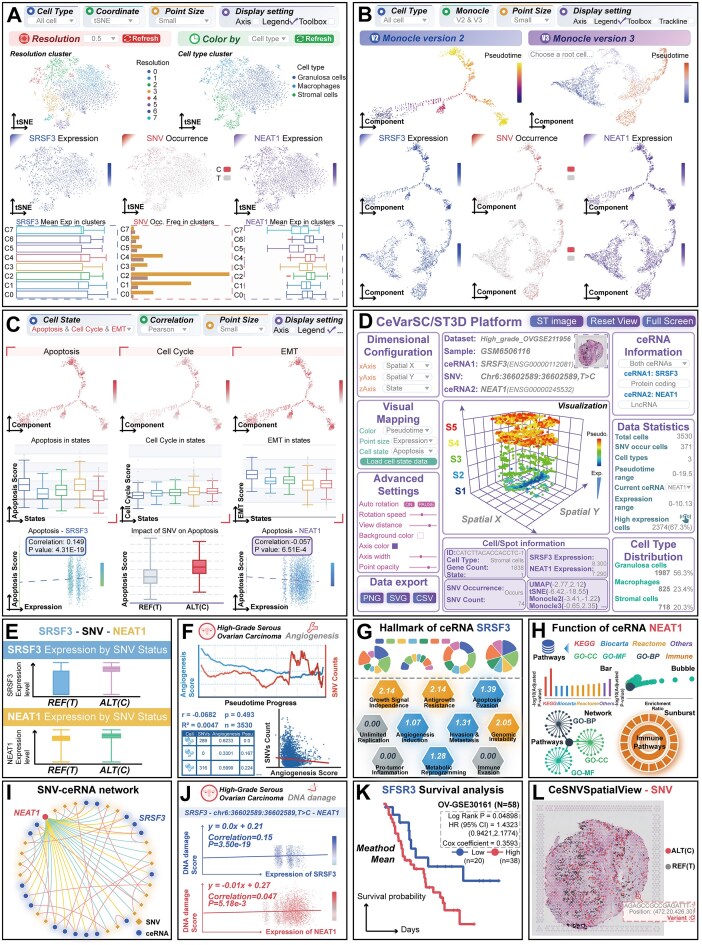
A case study for utilization of analysis tools in LnCeVar 2.0. (**A**) *CeVarCluster* maps *NEAT1*-related SNV–ceRNA events across cell populations, stratifying cells/spatial spots visualization by cell type, state, gene content. (**B**) *CeVarTraject* constructs and visualizes developmental cell trajectories to elucidate dynamics of *NEAT1*-related SNV–ceRNA events and their lineage associations. (**C**) *CeVarState* examines SNV–ceRNA impact on cellular function heterogeneity by assessing cell states (e.g. apoptosis, cell cycle, EMT) and their links to SNV genotypes/ceRNA expression across contexts. (**D**) *CeVarSC3D* and *CeVarST3D* enable multilevel cross talk analyses of SNVs, ceRNA networks, and cell states in disease pathology at single-cell/spatial levels via interactive 3D views. (**E**–**L**) A panel of mini tools sheds light on the regulatory role of SNVs in *NEAT1*-related ceRNAs, revealing potential therapeutic implications for clinical practice in HGSOC.

Moreover, to further characterize SNV–ceRNA events involving cellular heterogeneity and dynamic states within the high-grade serous ovarian carcinoma (HGSOC) microenvironment, thereby facilitating the dissection of intercellular interactions and signaling pathways, a panel of online tools has been developed to study ceRNA-regulated mechanisms at the single-cell and spatial levels (Fig. 3A–D). The *CeVarCluster* tool was used to investigate the comprehensive mapping of *NEAT1*-related SNV–ceRNA events across diverse cell populations (Fig. 3A). Within this tool, cells and spatial spots can be stratified into distinct clusters, enabling visualization of ceRNA expression profiles and SNV genotype occurrences based on cell type, cell state, gene content, and other relevant features (Supplementary Fig. S11). To elucidate the dynamics of *NEAT1*-related SNV–ceRNA events and their associations across different lineages, the *CeVarTraject* tool was used to construct and visualize the developmental cell trajectories (Fig. 3B and Supplementary Fig. S12). To explore the regulatory impact of SNV–ceRNA events on heterogeneity in cellular functions, the *CeVarState* tool was used to evaluate cell states (such as apoptosis, cell cycle, and epithelial mesenchymal transition) and their associations with SNV genotypes and ceRNA expression across diverse functional contexts (Fig. 3C and Supplementary Fig. S13). The *CeVarSC3D* and *CeVarST3D* tools perform multilevel cross talk analyses of SNVs, ceRNA networks, and cell states underlying individual disease pathology at the single-cell and spatial levels, presented in an interactive 3D view (Fig. 3D and Supplementary Fig. S14). These tools enable the examination of the complex interplay between *NEAT1*-associated ceRNA expression and genome variations, which influences individual cell functions and disease pathology.

Several mini tools offer quick and easy-to-use analyses, including functional, hallmark and cell state annotations, cell clustering, survival and correlation analyses, and SNV–ceRNA network construction (Fig. 3E–L). Using these tools, we found that distinct SNV genotypes modulate the ceRNA interaction between *NEAT1* and *SRSF3* in HGSOC cells (Fig. 3E). Furthermore, the cumulative burden of genome mutations is closely associated with HGSOC angiogenesis (Fig. 3F). Previous studies have demonstrated that *NEAT1* knockdown inhibits homologous recombination capacity and increases Olaparib-induced DNA damage in HGSOC, thereby enhancing their sensitivity to Olaparib and conferring a critical therapeutic advantage [42]. Additionally, *SRSF3* overexpression is essential for ovarian cancer cell growth and survival, while its knockdown impairs DNA repair activity, with underlying mechanisms explored in prior research [43]. Using the analysis tools in LnCeVar 2.0, we revealed that the *NEAT1–SRSF3* interaction is involved in key biological processes driving cancer initiation and progression, such as genome instability and mutation (Fig. 3G–I). We also observed a positive correlation between *NEAT1–SRSF3* expression levels and DNA damage (Fig. 3J) and found that high *SRSF3* expression is associated with poor prognosis in HGSOC (Fig. 3K). These findings, integrating existing knowledge with novel insights derived from LnCeVar 2.0, unraveling the roles of SNV–ceRNA networks in disease pathology, thereby facilitating a deeper understanding of ovarian cancer mechanisms. The spatial map of ceRNA expression and SNV mutations enables visualization and investigation of their impact on disease characteristics through integration with spatial pathological section data (Fig. 3L). In summary, the web-based tools of LnCeVar 2.0 provide efficient means of analyzing SNV–ceRNA regulatory mechanisms, facilitating detailed integration, comparison, and visualization.

## Discussion

The development of LnCeVar 2.0 represents a significant advancement in the field of ceRNA and SNV research, addressing the growing demands posed by the expansion of single-cell and spatial transcriptomics datasets. By integrating a vast array of high-throughput scRNA-seq, stRNA-seq, and bulk-seq data across over 20 000 normal and disease samples from human and mouse, this updated database provides an enhanced resource for investigating SNV and gene expression regulation at cellular and spatial resolutions. The inclusion of over 1.7 million cells, 900 thousand spatial spots, and 400 distinct cell types enables comprehensive analyses of the tumor microenvironment and healthy tissue landscapes, facilitating comparative studies that were previously constrained by data limitations.

Compared to LnCeVar 1.0, LnCeVar 2.0 clarifies the mechanistic links between SNVs, ceRNA dynamics, and cell fate decisions, particularly in the context of disease progression and treatment responses. For instance, it identifies *NEAT1*-related SNV–ceRNA events as potential targets by linking them to cancer hallmarks (e.g. genomic instability) and drug sensitivity (e.g. *NEAT1* enhancing HGSOC’s response to Olaparib). It addresses traditional single-gene target limitations via multidimensional data integration, aiding targeted therapy development and overcoming drug resistance. Although LnCeVar 2.0 has identified a large number of SNV–ceRNA events, it is limited in that only a small proportion of these events have been experimentally validated and found to contribute to the development of specific diseases. Many of the remaining events require further analysis and validation. LnCeVar 2.0 will continuously improve research methods and tools for analysing candidate SNV–ceRNA events to address this limitation.

Looking to the future, LnCeVar 2.0 will continue to expand its data coverage, focusing on integrating multimodal datasets to capture the full complexity of genome variation and gene regulation. By bridging genome variations, single-cell and spatial dynamics, and clinical outcomes, LnCeVar 2.0 ultimately aims to accelerate discoveries in disease mechanisms and facilitate the development of personalized therapeutic strategies.

## Supplementary Material

gkaf1009_Supplemental_File

## Data Availability

All the data can be downloaded from http://bio-bigdata.hrbmu.edu.cn/LnCeVar or http://www.bio-bigdata.net/LnCeVar.
