# Supplementary material for: LnCeVar 2.0: an updated resource and web tools for genomic variations disrupting ceRNA networks from single-cell/spatial transcriptomics data
Source: Nucleic Acids Res. 2025 Oct 16;54(D1):D194–203. doi: 10.1093/nar/gkaf1009 (PMC12807714; doi:10.1093/nar/gkaf1009)
Supplement: gkaf1009_Supplemental_File [file gkaf1009_supplemental_file.pdf]

Supplementary Information for

LnCeVar 2.0: an updated resource and web tools for genomic variations disrupting ceRNA networks from single-cell/spatial transcriptomics data

This file includes:

Supplementary Methods

High-throughput sequencing data collection

Data preprocessing and normalization

Cell clustering and visualization

Cell/spot type annotation

Cell development trajectories construction

Identification of functional single-cell and spatially specific SNV-ceRNA events

Functional annotation data collection

Manual curation of validated SNV-ceRNA events and biomarker annotations

Survival analysis of ceRNA regulations

Database construction

References of Supplementary Methods

Supplementary Figures (Figure S1-S14)

Supplementary Methods

High-throughput sequencing data collection

To develop a comprehensive database integrating high-throughput single-cell RNA sequencing (scRNA-seq) and spatial transcriptomics (stRNA-seq) datasets, LnCeVar 2.0 systematically collected and processed relevant data from multiple public sources. First, we conducted keyword-based literature searches on PubMed to manually select studies aligned with our research focus, then retrieved associated tissue information and metadata from the NCBI Gene Expression Omnibus (GEO; https://www.ncbi.nlm.nih.gov/geo/). Beyond literature-derived data, we integrated information from existing databases including TISCH2 (http://tisch.compbio.cn/home/) (1), DISCO (https://disco.bii.a-star.edu.sg/) (2), SpatialDB (http://spatialomics.org/SpatialDB/) (3), CROST (https://ngdc.cncb.ac.cn/crost/home/) (4) and ExMdb (http://www.bio-server.cn/ExMdb/) (5), which cover both single-cell and spatial transcriptomics. To ensure consistency for subsequent analyses, raw sequencing data were obtained from the NCBI Sequence Read Archive (SRA) and ArrayExpress (6), and underwent uniform preprocessing. Additionally, we incorporated multiple high-quality datasets officially released by 10x Genomics (https://www.10xgenomics.com/datasets) to further support the study.

Data preprocessing and normalization

All raw FASTQ files were processed using pipelines developed by 10x Genomics for alignment and quantification: scRNA-seq libraries were processed with Cell Ranger (v9.0.1), while spatial transcriptome libraries were processed with Space Ranger (v3.1.3). Only datasets

containing more than 100 single cells or spatial spots were included. For each dataset, corresponding metadata, including sample IDs, organ/tissue origin, clinical treatment, biosample groups, primary/metastatic sites, and cell types, were integrated into LnCeVar 2.0. Gene annotation files were obtained from the GENCODE database (https://www.gencodegenes.org/; version GRCh38 for humans and GRCm39 for mouse) (7) to identify various gene types, such as protein-coding genes, long non-coding RNAs (lncRNAs), pseudogenes, and others. Downstream quality control and normalization of all transcriptomic profiles were conducted in R (v4.2.3) using Seurat (v4.3) (8). Briefly, raw count matrices were imported as Seurat objects. Cells with fewer than 100 detected genes or more than 10% mitochondrial reads were removed, and genes detected in fewer than three cells were excluded. The retained counts were normalized using Seurat's LogNormalize method (scaling by total counts per cell followed by log-transformation). For spatial transcriptomics, low-quality spots and lowly expressed genes were similarly excluded, and the filtered matrix was normalized using the Seurat SCTransform function.

Cell clustering and visualization

We performed unsupervised clustering of gene expression profiles using Seurat (v4.3). First, we applied principal component analysis (PCA) for dimensionality reduction and then selected a subset of informative principal components (PCs) based on inflection points identified via Seurat's ElbowPlot function. These PCs served as input for downstream clustering and visualization. Cell clustering was conducted using the FindClusters function, with the resolution parameter systematically varied from 0.1 to 0.9 (in 0.1 increments) to generate

results across a range of granularities; higher resolution values yielded finer cluster distinctions. For visualizing cellular heterogeneity, we used both t-distributed stochastic neighbor embedding (RunTSNE) and uniform manifold approximation and projection (RunUMAP) on the selected PCs. This multi-resolution strategy enabled robust exploration of clustering stability and biological interpretability.

Cell/spot type annotation

LnCeVar 2.0 employs two strategies for cell type annotation: *(i)* using original cell type annotations provided by data sources; and *(ii)* applying the CELLiD method as described in DISCO (2). In this step, cell types for each cluster were determined using reference cell type marker genes and the CELLiD R code (https://disco.bii.a-star.edu.sg/tool/cellid). Cell marker annotations were integrated from DISCO and CellMarker 2.0 (9). DISCO is a database with deeply integrated scRNA-seq data spanning hundreds of tissues, cell lines, organoids, and diseases, while CellMarker 2.0 offers manually curated markers for diverse cell types. Markers from both databases were combined to enable comprehensive cell type annotations. CELLiD analysis of each cell cluster was performed using a comprehensive reference for cell type annotation, which encompasses normal cells, malignant cells, and disease-associated cells.

Cell development trajectories construction

We used the R packages Monocle 2 (v2.18.0) (10) and Monocle 3 (v1.2.9) (11) to calculate pseudotime and cell states, and to construct cell developmental trajectories. Monocle 2 utilizes gene counts as the input matrix and performs well with both relative expression data and

count-based measures. The expressionFamily parameter was set to negbinomial.size() to specify the negative binomial distribution. Processed data from Seurat, including quality-filtered cells and metadata, were used for the Monocle 2 analysis. For the principal component analysis (PCA), we included genes with an average expression greater than 0.1, and used the top 20 principal components for cell clustering. Differentially expressed genes (q-value < 0.01) were selected for cell ordering. The DDRTree algorithm was then applied for dimensionality reduction to visualize trajectories in two-dimensional space. Monocle 3 employs UMAP for dimensionality reduction, which more effectively captures high-dimensional data characteristics. Unlike Monocle 2's DDRTree-based method, which assumes a single tree-like trajectory structure, Monocle 3 can model multiple disjoint trajectory graphs. In Monocle 3 analysis, the top 20 principal components from PCA were used for cell clustering, and genes with the highest expression (q-value < 0.01) were selected for cell ordering. Pseudotime trajectories were visualized in two-dimensional UMAP space.

Identification of functional single-cell and spatially specific SNV-ceRNA events

To identify single-nucleotide variants (SNVs) at single-cell and spatial resolutions, we implemented modality-specific pipelines. For scRNA-seq data, raw fastq files were processed using scSNV (12), with the algorithm's default barcode whitelist replaced by the latest 10x Genomics barcode inclusion list to ensure compatibility with current library chemistries and improve cell recovery. For spatial transcriptomics data, we used SComatic (13), a single-cell resolution SNV detection algorithm. Inputs included Space Ranger-derived BAM files and spatial spot cluster assignments, and SComatic was run with default parameters to detect

spot-specific SNVs across tissue sections.

LnCeVar 2.0 compiles ceRNA candidates from existing databases, including LnCeCell 2.0, LncACTdb 3.0, and starBase 2.0, providing information on potential ceRNA interactions at single-cell and spatial resolutions (14-16). A probability theory-based method that had previously been published was applied to construct cell-specific networks and identify ceRNAs at these resolutions (17). For each ceRNA interaction, P-values were calculated to test the statistical independence of the candidates within individual cells or spots. A ceRNA activity score was then computed as -log10 (P-value). To evaluate the disruptive effects of SNVs on ceRNA regulation, TargetScan (v.6.0) (18) was used to identify miRNA-gene interactions and assess the impact of genomic variations on microRNA-binding sites. SNVs were then mapped to the miRNA binding sites of each ceRNA pair based on genomic locations. A candidate SNV-ceRNA event was defined as a situation in which different genotypes of a variant altered ceRNA regulation (e.g. gain or loss of, or alteration to, miRNA binding sites). To further identify functional SNV-ceRNA events at the expression level, a multivariate multiple regression (MMR) model was employed to examine whether a given SNV regulates the expression of both genes in a ceRNA pair (19-21). In LnCeVar 2.0, the effect of functional events is estimated using SNV-specific coefficients (Coef values), while model significance (P-values) is assessed via Pillai's trace statistics. For example, in a typical SNV-ceRNA event (structured as ceRNA1-SNV-ceRNA2), the MMR model evaluates the SNV's regulatory effects on ceRNA1 and ceRNA2, these effects are quantified as coef1 and coef2, respectively. Correspondingly, the significance of the model's predictions for ceRNA1 and ceRNA2 is denoted as P1 and P2.

Functional annotation data collection

LnCeVar 2.0 incorporates a large number of functional gene sets to analyze the functional activation status and state transitions of individual cellular populations, with 18,100 sets for the human genome and 14,039 for the mouse genome. These gene sets cover diverse functional annotations, including Gene Ontology (GO) (22), canonical pathways (23), cancer cell states (24), and classical cancer hallmarks (25), thereby facilitating comprehensive functional analysis. Notably, it includes 10 classic cancer hallmark gene sets, representing well-defined biological processes such as self-sufficiency in growth signals, insensitivity to antigrowth signals, evasion of apoptosis, limitless replicative potential, sustained angiogenesis, tissue invasion and metastasis, genome instability and mutation, tumor-promoting inflammation, reprogramming energy metabolism, and evasion of immune detection. Additionally, 14 characteristic gene sets for functional states of cancer cells (e.g., stemness, invasion, metastasis, proliferation, EMT, angiogenesis, apoptosis, cell cycle, differentiation, DNA damage, DNA repair, hypoxia, inflammation, and quiescence) are included, sourced from the CancerSEA database (24).

LnCeVar 2.0 employs a hypergeometric test for the evaluation of significant enrichment in distinct functional contexts. If there are a total of N genes in the genome, of which S is involved in the gene set under investigation, and there are a total of M interesting target genes for analysis, of which x are involved with the same function, then the P value can be calculated as:

$$P = 1 - \sum_{t=0}^{x} \frac{\binom{S}{t}\binom{N-S}{M-t}}{\binom{N}{M}} \qquad (1)$$

Significantly enriched functions were defined at a level of P < 0.05 and were further illustrated as a bar graph of the -log10 (P-value).

Manual curation of validated SNV-ceRNA events and biomarker annotations

To provide more detailed and reliable information regarding the mechanisms of SNV and ceRNA regulation, LnCeVar 2.0 has been updated to include an expanded set of experimentally verified ceRNA interactions and SNV-ceRNA events, which were collated from our previous studies and manual curation (14-16,26-28). To collect high-confidence variation-ceRNA associations, we retrieved published literature from PubMed related to SNPs, somatic mutations, copy number variations (CNV) and ceRNAs. We searched the PubMed database for relevant articles using the following combination of keywords: "miRNA sponge" OR "ceRNA" OR "miRNA decoy" OR "competing RNA" OR "antagomir" OR "miRNA mediated" AND "SNP" OR "mutation" OR "CNV". These candidate articles were reviewed by at least two researchers. A total of 5,785 validated ceRNA interactions and SNV-ceRNA events were confirmed using high-confidence experimental techniques, including PCR, Western blotting, luciferase reporter assays and other methodologies. Additionally, LnCeVar 2.0 conducted manual curation of ceRNA and SNV annotations related to cancer biomarkers associated with disease pathology, diagnosis, and treatment. We used the following keyword combinations: ("circulating" OR "drug-resistant" OR "prognostic" OR "immune" OR "metastasis" OR "recurrence" OR "cell growth" OR "EMT" OR "apoptosis" OR "autophagy") AND ("ceRNA") to collect diagnostic and therapeutic biomarkers. Biomarkers were included if a ceRNA regulation was linked to these processes via overexpression, RNA knockdown, or other functional experiments. A total of 16,937 gene biomarkers associated with drug resistance, circulation, survival, immunity, metastasis, recurrence, cell growth, epithelial-mesenchymal transition

(EMT), apoptosis and autophagy were manually curated.

Survival analysis of ceRNA regulations

LnCeVar 2.0 integrated bulk-seq data and clinical profiles from 22,515 tumour samples across various types. These datasets, sourced from TCGA (29) and NCBI-GEO (30), encompass ceRNA expression, genome variation across individuals, clinicopathological features, clinical treatment details, and follow-up data. Cox regression analysis was performed to evaluate the association between survival state and the expression level of each gene in a ceRNA interaction.

Database construction

The LnCeVar 2.0 database can be accessed at: http://bio-bigdata.hrbmu.edu.cn/LnCeVar or http://www.bio-bigdata.net/LnCeVar. The previous version (LnCeVar 1.0, available at http://www.bio-bigdata.net/LnCeVar1.0/) remains accessible for users requiring legacy data or features. The online web server of LnCeVar 2.0 was constructed using Java Server Pages (JSP) and deployed on Tomcat software (v6, https://tomcat.apache.org/). The web pages were created using HyperText Markup Language (HTML) and controlled by Java programs (https://www.oracle.com/java/). All datasets of LnCeVar 2.0 were documented and managed using the MySQL database server (version 5.5, http://www.mysql.com). Several JavaScript libraries were integrated to enable interactive data visualization and table rendering, including jQuery (version 1.11.3, https://code.jquery.com), Datatables (version 1.10.10, http://www.datatables.club/) and ECharts.js (version 4.0, https://echarts.apache.org/).

References of Supplementary Methods

1. Han, Y., Wang, Y., Dong, X., Sun, D., Liu, Z., Yue, J., Wang, H., Li, T. and Wang, C. (2023) TISCH2: expanded datasets and new tools for single-cell transcriptome analyses of the tumor microenvironment. *Nucleic Acids Res*, **51**, D1425-D1431.
2. Li, M., Zhang, X., Ang, K.S., Ling, J., Sethi, R., Lee, N.Y.S., Ginhoux, F. and Chen, J. (2022) DISCO: a database of Deeply Integrated human Single-Cell Omics data. *Nucleic Acids Res*, **50**, D596-D602.
3. Fan, Z., Chen, R. and Chen, X. (2020) SpatialDB: a database for spatially resolved transcriptomes. *Nucleic Acids Res*, **48**, D233-D237.
4. Wang, G., Wu, S., Xiong, Z., Qu, H., Fang, X. and Bao, Y. (2024) CROST: a comprehensive repository of spatial transcriptomics. *Nucleic Acids Res*, **52**, D882-D890.
5. Qi, Y., Xu, R., Song, C., Hao, M., Gao, Y., Xin, M., Liu, Q., Chen, H., Wu, X., Sun, R. *et al.* (2024) A comprehensive database of exosome molecular biomarkers and disease-gene associations. *Sci Data*, **11**, 210.
6. Athar, A., Fullgrabe, A., George, N., Iqbal, H., Huerta, L., Ali, A., Snow, C., Fonseca, N.A., Petryszak, R., Papatheodorou, I. *et al.* (2019) ArrayExpress update - from bulk to single-cell expression data. *Nucleic Acids Res*, **47**, D711-D715.
7. Frankish, A., Carbonell-Sala, S., Diekhans, M., Jungreis, I., Loveland, J.E., Mudge, J.M., Sisu, C., Wright, J.C., Arnan, C., Barnes, I. *et al.* (2023) GENCODE: reference annotation for the human and mouse genomes in 2023. *Nucleic Acids Res*, **51**, D942-D949.
8. Satija, R., Farrell, J.A., Gennert, D., Schier, A.F. and Regev, A. (2015) Spatial reconstruction of single-cell gene expression data. *Nat Biotechnol*, **33**, 495-502.
9. Hu, C., Li, T., Xu, Y., Zhang, X., Li, F., Bai, J., Chen, J., Jiang, W., Yang, K., Ou, Q. *et al.* (2023) CellMarker 2.0: an updated database of manually curated cell markers in human/mouse and web tools based on scRNA-seq data. *Nucleic Acids Res*, **51**, D870-D876.
10. Qiu, X., Mao, Q., Tang, Y., Wang, L., Chawla, R., Pliner, H.A. and Trapnell, C. (2017) Reversed graph embedding resolves complex single-cell trajectories. *Nat Methods*, **14**, 979-982.
11. Cao, J., Spielmann, M., Qiu, X., Huang, X., Ibrahim, D.M., Hill, A.J., Zhang, F., Mundlos, S., Christiansen, L., Steemers, F.J. *et al.* (2019) The single-cell transcriptional landscape of mammalian organogenesis. *Nature*, **566**, 496-502.
12. Wilson, G.W., Derouet, M., Darling, G.E. and Yeung, J.C. (2021) scSNV: accurate dscRNA-seq SNV co-expression analysis using duplicate tag collapsing. *Genome Biol*, **22**, 144.
13. Muyas, F., Sauer, C.M., Valle-Inclan, J.E., Li, R., Rahbari, R., Mitchell, T.J., Hormoz, S. and Cortes-Ciriano, I. (2024) De novo detection of somatic mutations in high-throughput single-cell profiling data sets. *Nat Biotechnol*, **42**, 758-767.
14. Wang, P., Li, X., Gao, Y., Guo, Q., Wang, Y., Fang, Y., Ma, X., Zhi, H., Zhou, D., Shen, W. *et al.* (2019) LncACTdb 2.0: an updated database of experimentally supported ceRNA interactions curated from low- and high-throughput experiments. *Nucleic Acids Res*, **47**, D121-D127.
15. Li, J.H., Liu, S., Zhou, H., Qu, L.H. and Yang, J.H. (2014) starBase v2.0: decoding miRNA-ceRNA, miRNA-ncRNA and protein-RNA interaction networks from large-scale CLIP-Seq data. *Nucleic Acids Res*, **42**, D92-97.
16. Guo, Q., Liu, Q., He, D., Xin, M., Dai, Y., Sun, R., Li, H., Zhang, Y., Li, J., Kong, C. *et al.* (2024) LnCeCell 2.0: an updated resource for lncRNA-associated ceRNA networks and web tools based on single-cell and spatial transcriptomics sequencing data. *Nucleic Acids Res*.

17. Dai, H., Li, L., Zeng, T. and Chen, L. (2019) Cell-specific network constructed by single-cell RNA sequencing data. *Nucleic Acids Res*, **47**, e62.
18. Friedman, R.C., Farh, K.K., Burge, C.B. and Bartel, D.P. (2009) Most mammalian mRNAs are conserved targets of microRNAs. *Genome Res*, **19**, 92-105.
19. Xu, R., He, D., Sun, R., Zhou, J., Xin, M., Liu, Q., Dai, Y., Li, H., Zhang, Y., Li, J. *et al.* (2025) CNV-mediated dysregulation of the ceRNA network mechanism revealed heterogeneity in diffuse and intestinal gastric cancers. *J Transl Med*, **23**, 308.
20. Wang, P., Li, X., Gao, Y., Guo, Q., Ning, S., Zhang, Y., Shang, S., Wang, J., Wang, Y., Zhi, H. *et al.* (2020) LnCeVar: a comprehensive database of genomic variations that disturb ceRNA network regulation. *Nucleic Acids Res*, **48**, D111-D117.
21. Li, M.J., Zhang, J., Liang, Q., Xuan, C., Wu, J., Jiang, P., Li, W., Zhu, Y., Wang, P., Fernandez, D. *et al.* (2017) Exploring genetic associations with ceRNA regulation in the human genome. *Nucleic Acids Res*, **45**, 5653-5665.
22. Gene Ontology, C. (2021) The Gene Ontology resource: enriching a GOld mine. *Nucleic Acids Res*, **49**, D325-D334.
23. Castanza, A.S., Recla, J.M., Eby, D., Thorvaldsdottir, H., Bult, C.J. and Mesirov, J.P. (2023) Extending support for mouse data in the Molecular Signatures Database (MSigDB). *Nat Methods*, **20**, 1619-1620.
24. Yuan, H., Yan, M., Zhang, G., Liu, W., Deng, C., Liao, G., Xu, L., Luo, T., Yan, H., Long, Z. *et al.* (2019) CancerSEA: a cancer single-cell state atlas. *Nucleic Acids Res*, **47**, D900-D908.
25. Hanahan, D. and Weinberg, R.A. (2011) Hallmarks of cancer: the next generation. *Cell*, **144**, 646-674.
26. Wang, P., Guo, Q., Hao, Y., Liu, Q., Gao, Y., Zhi, H., Li, X., Shang, S., Guo, S., Zhang, Y. *et al.* (2021) LnCeCell: a comprehensive database of predicted lncRNA-associated ceRNA networks at single-cell resolution. *Nucleic Acids Res*, **49**, D125-D133.
27. Wang, P., Guo, Q., Qi, Y., Hao, Y., Gao, Y., Zhi, H., Zhang, Y., Sun, Y., Zhang, Y., Xin, M. *et al.* (2022) LncACTdb 3.0: an updated database of experimentally supported ceRNA interactions and personalized networks contributing to precision medicine. *Nucleic Acids Res*, **50**, D183-D189.
28. Gao, Y., Shang, S., Guo, S., Li, X., Zhou, H., Liu, H., Sun, Y., Wang, J., Wang, P., Zhi, H. *et al.* (2021) Lnc2Cancer 3.0: an updated resource for experimentally supported lncRNA/circRNA cancer associations and web tools based on RNA-seq and scRNA-seq data. *Nucleic Acids Res*, **49**, D1251-D1258.
29. Carrot-Zhang, J., Chambwe, N., Damrauer, J.S., Knijnenburg, T.A., Robertson, A.G., Yau, C., Zhou, W., Berger, A.C., Huang, K.L., Newberg, J.Y. *et al.* (2020) Comprehensive Analysis of Genetic Ancestry and Its Molecular Correlates in Cancer. *Cancer Cell*, **37**, 639-654 e636.
30. Barrett, T., Wilhite, S.E., Ledoux, P., Evangelista, C., Kim, I.F., Tomashevsky, M., Marshall, K.A., Phillippy, K.H., Sherman, P.M., Holko, M. *et al.* (2013) NCBI GEO: archive for functional genomics data sets--update. *Nucleic Acids Res*, **41**, D991-995.

Supplementary Figures

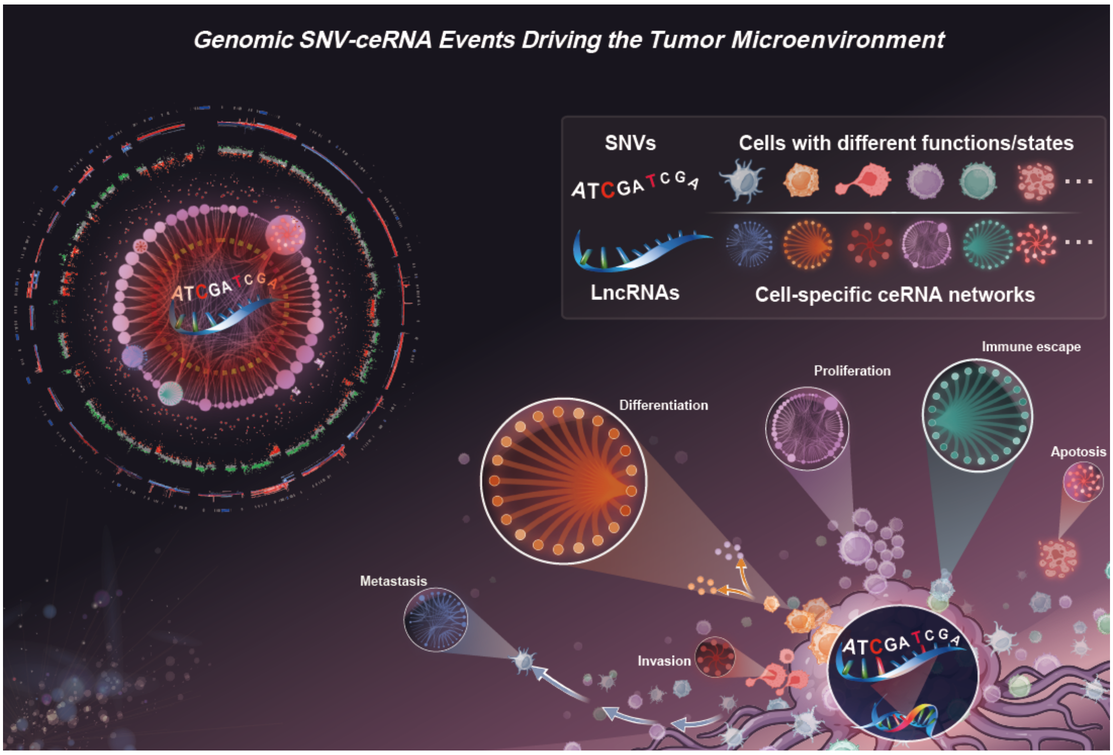

Figure S1. Schematic diagram of the background mechanism of LnCeVar 2.0. This diagram illustrates the core mechanism framework of LnCeVar 2.0, encompassing a comprehensive profile of SNVs in human and mouse genomes, as well as their regulatory effects on gene expression at single-cell and spatial levels by disrupting ceRNA networks.

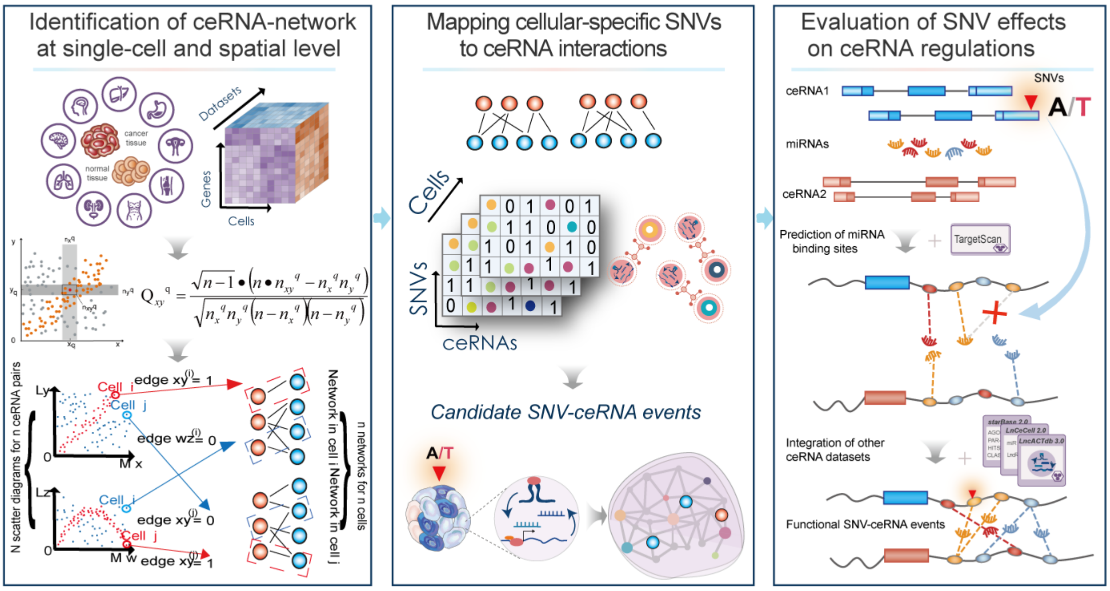

Figure S2. The pipeline for identifying functional single-cell and spatially specific SNV-ceRNA events.

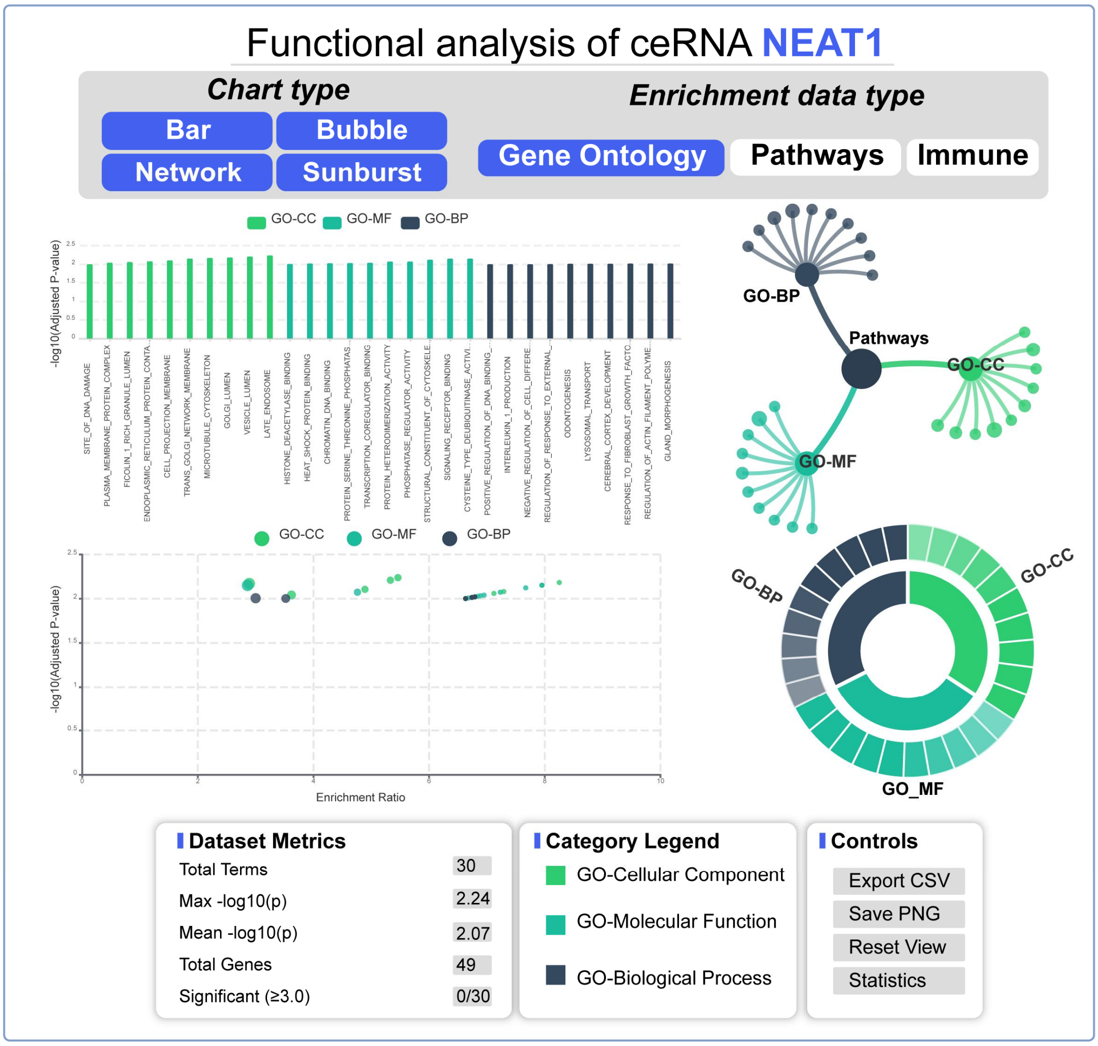

Figure S3. An overview of the functional annotations integrated into LnCeVar 2.0. These annotations facilitate a comprehensive functional analysis of the functional activation status and state transitions of cellular populations.

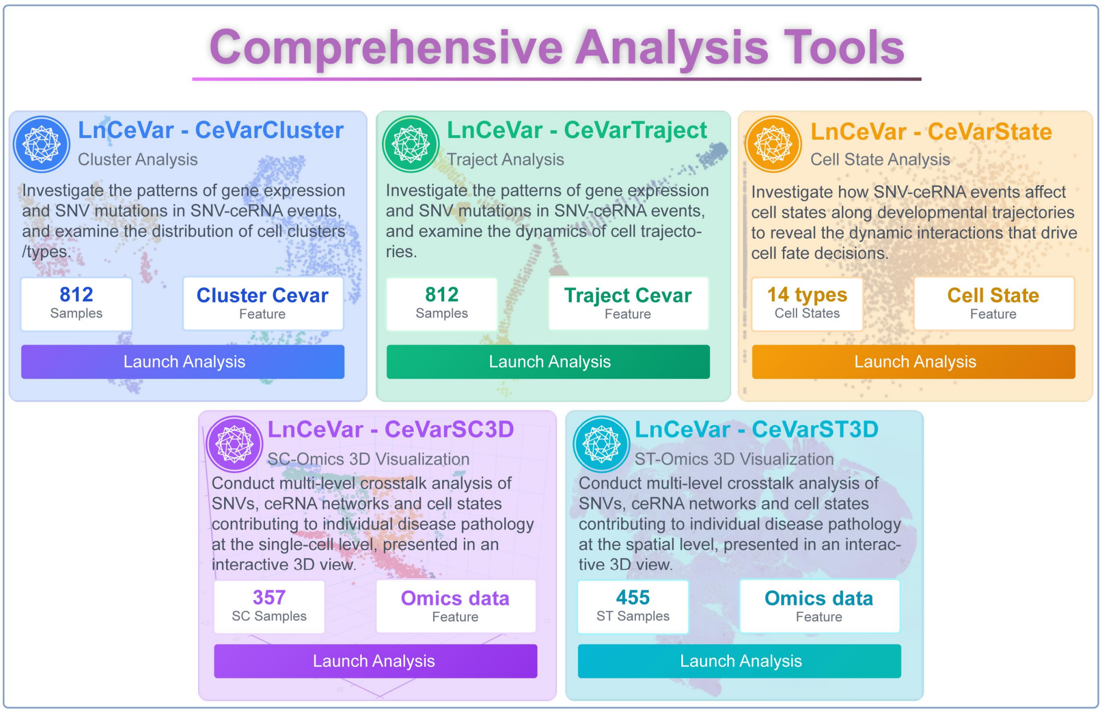

Figure S4. A panel of integrated analysis tools has been developed to enable in-depth investigation of SNV-ceRNA regulatory mechanisms at both single-cell and spatial levels.

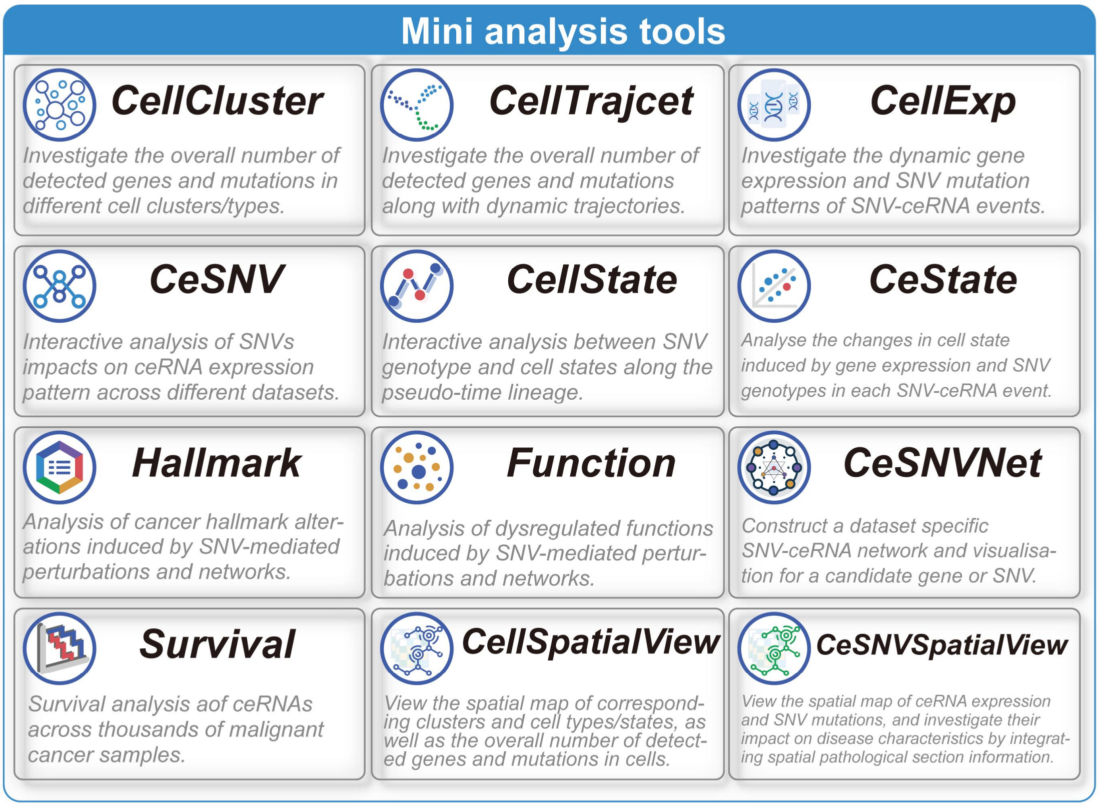

Figure S5. A panel of 12 mini-tools enables fast, user-friendly analyses, including functional annotations, cancer hallmark annotations, cell state annotations, cell clustering, survival analyses, correlation analyses, and SNV-ceRNA network construction.

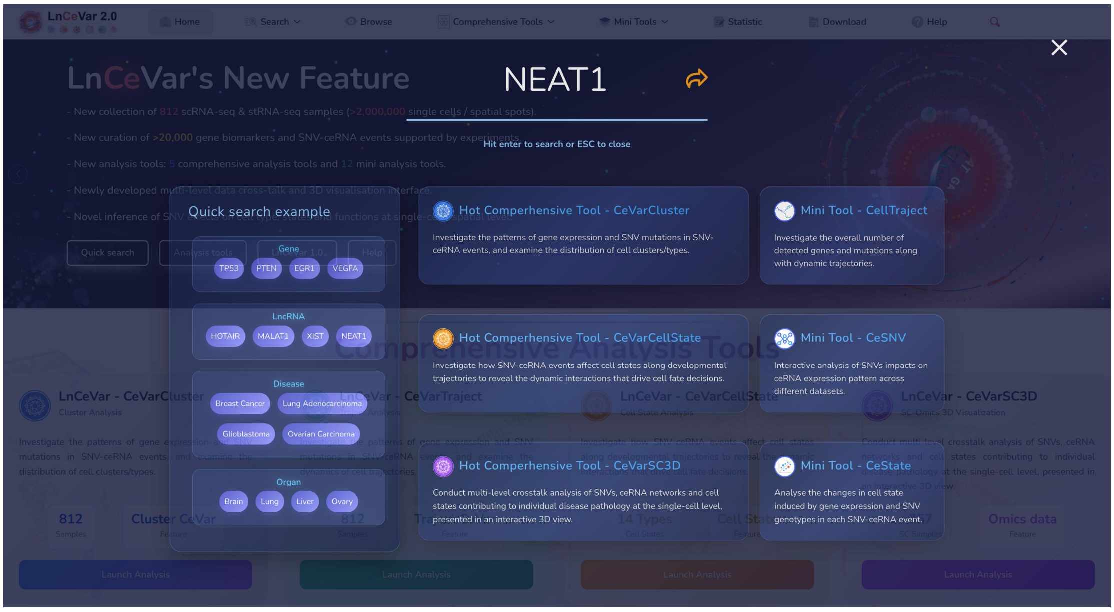

Figure S6. A screenshot of the 'QUICK SEARCH' page in LnCeVar 2.0, using the lncRNA *NEAT1* as an example.

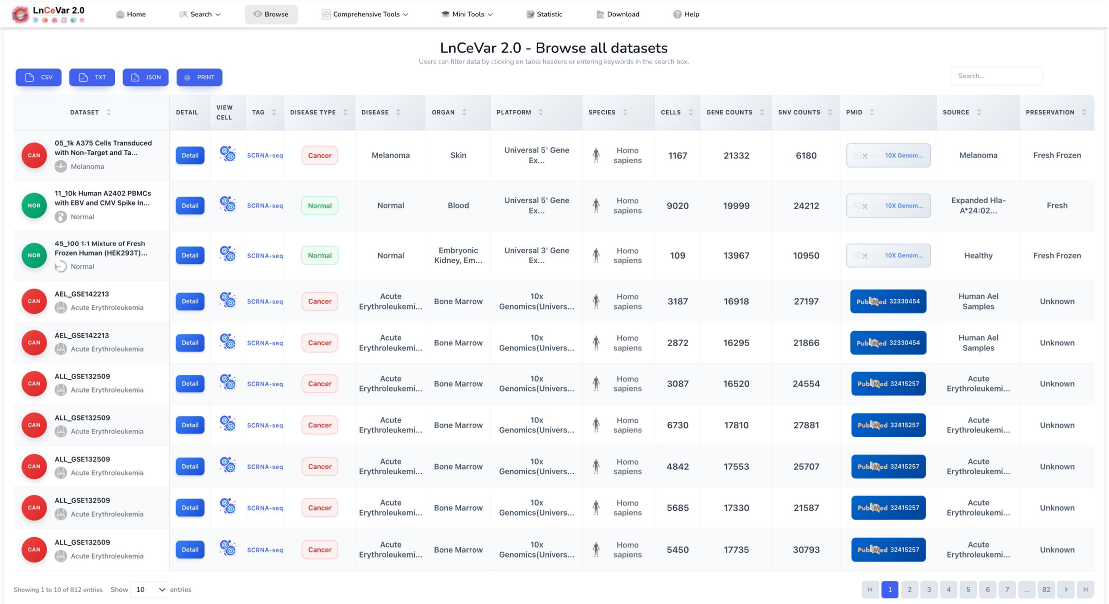

Figure S7. A screenshot of the 'Browse' page in LnCeVar 2.0, using the lncRNA *NEAT1* as an example.

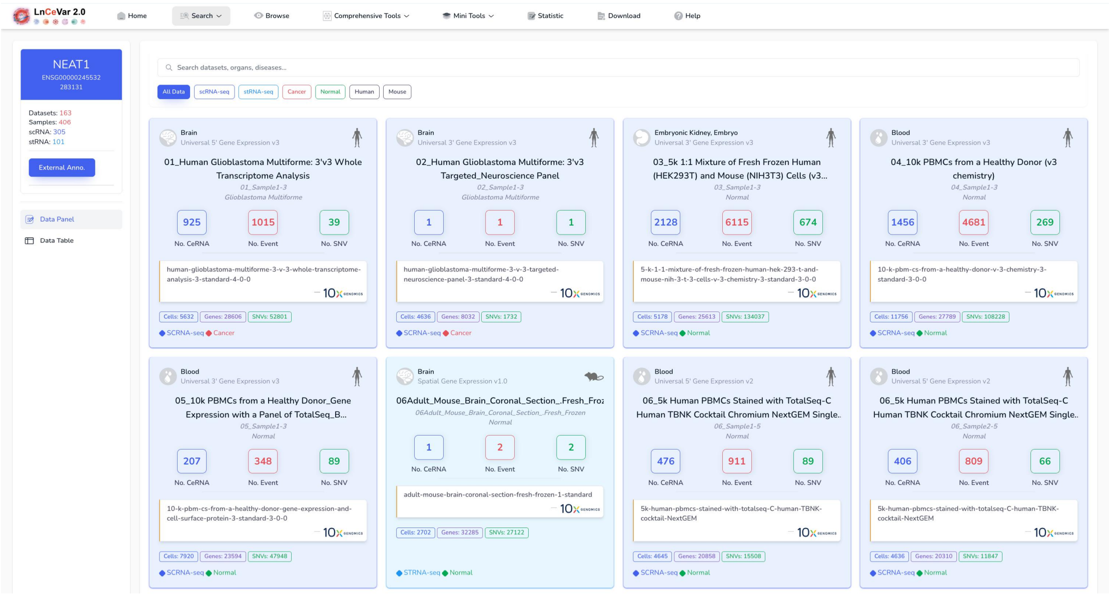

Figure S8. Data panels related to *NEAT1* as illustrated by LnCeVar 2.0.

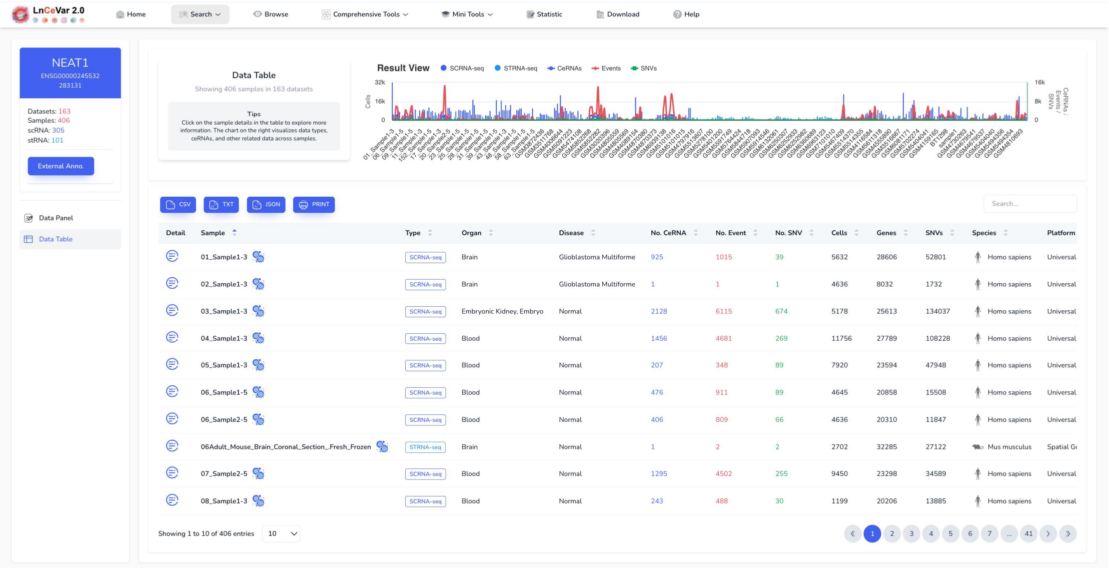

Figure S9. A data table listing information related to *NEAT1* as provided by LnCeVar 2.0.

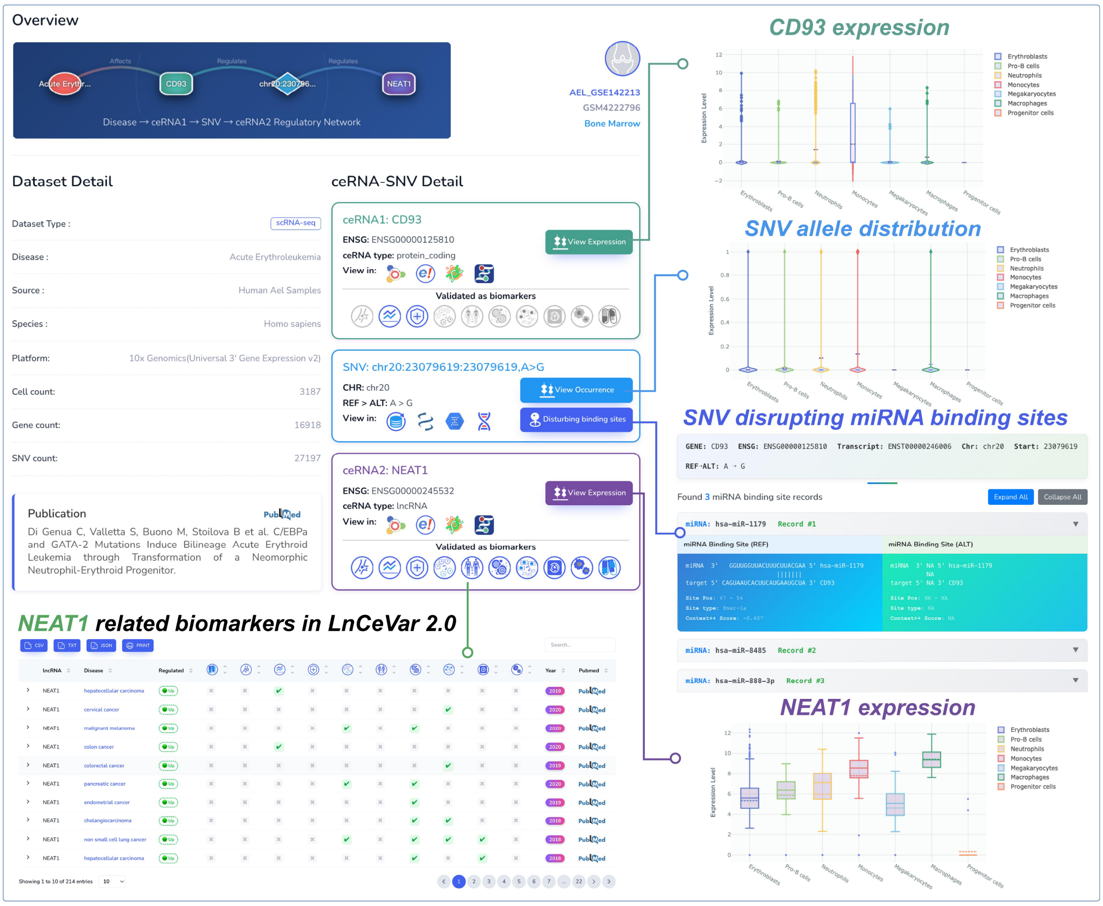

Figure S10. The detailed SNV-ceRNA information page in LnCeVar 2.0. This page contains comprehensive information on disease-SNV-ceRNA associations, showcasing how different SNV genotypes influence ceRNA regulatory states and further impact downstream gene expression across single-cell and spatial omics datasets from various diseases.

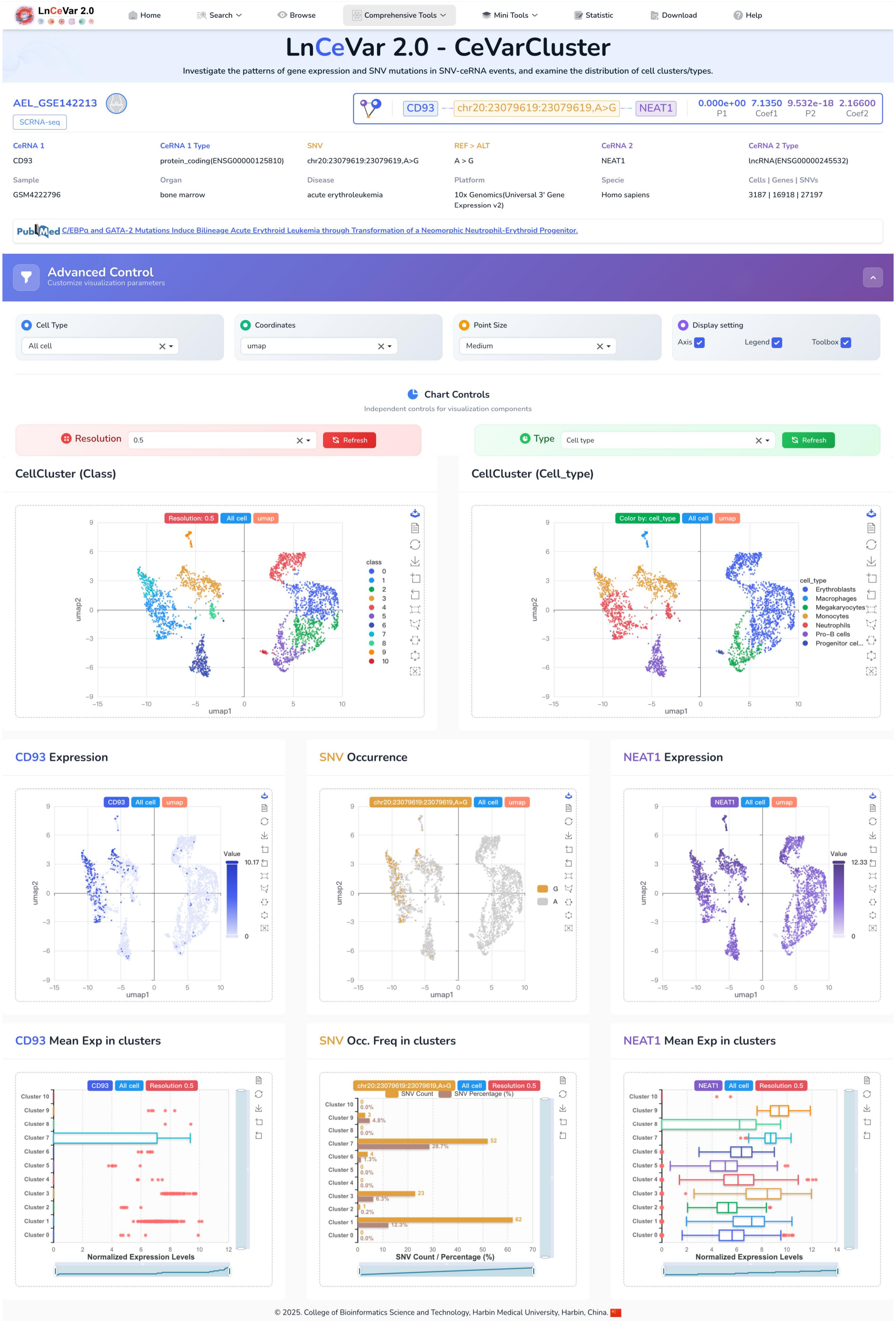

Figure S11. The *CeVarCluster* tool for mapping *NEAT1*-related SNV-ceRNA events across cell populations. It stratifies cells and spatial spots into distinct clusters, enabling visualization of ceRNA expression profiles and SNV genotypes by cell type, state, and other features.

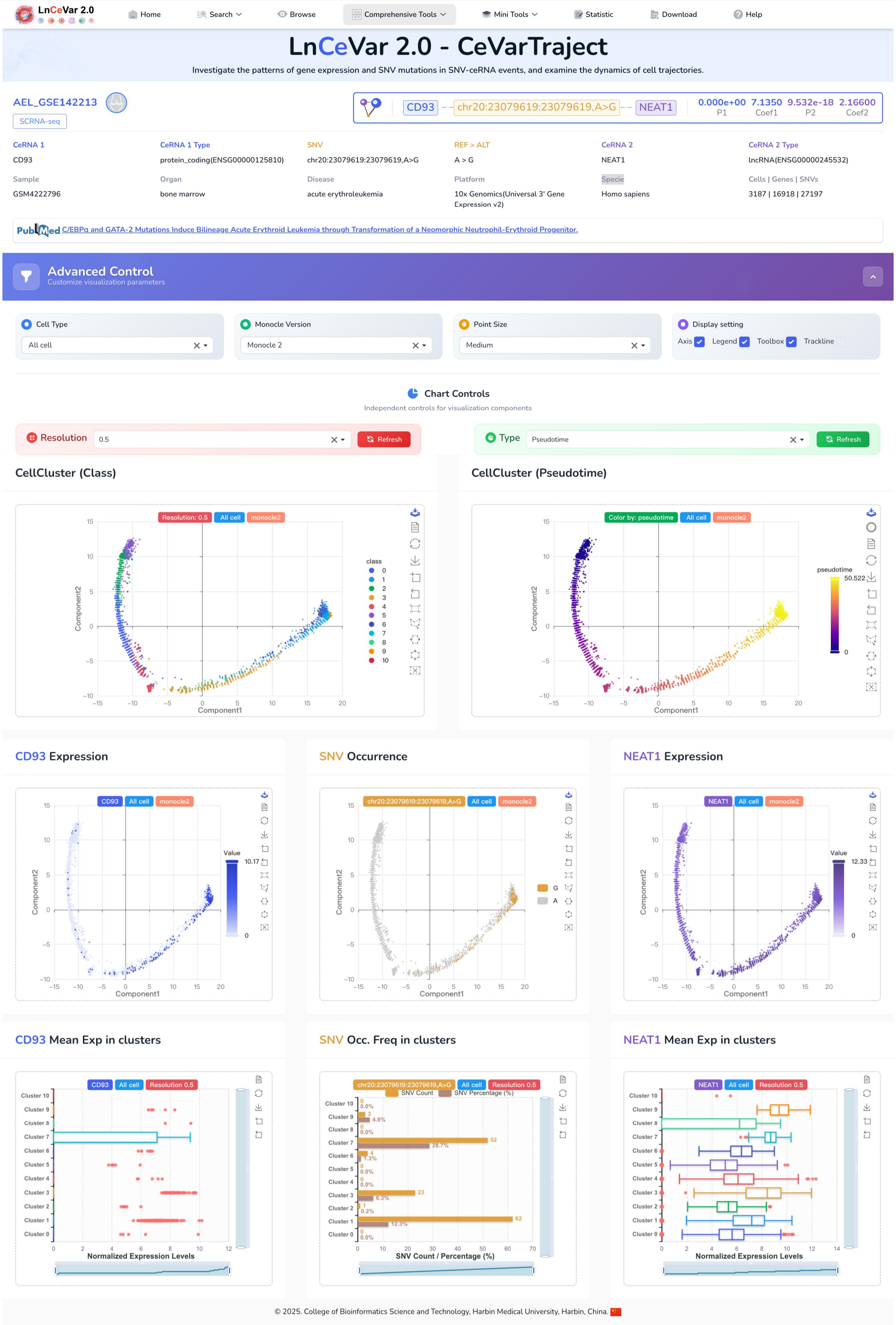

Figure S12. The *CeVarTraject* tool for elucidating dynamics of *NEAT1*-related SNV-ceRNA events and their lineage associations by constructing and visualizing developmental cell trajectories.

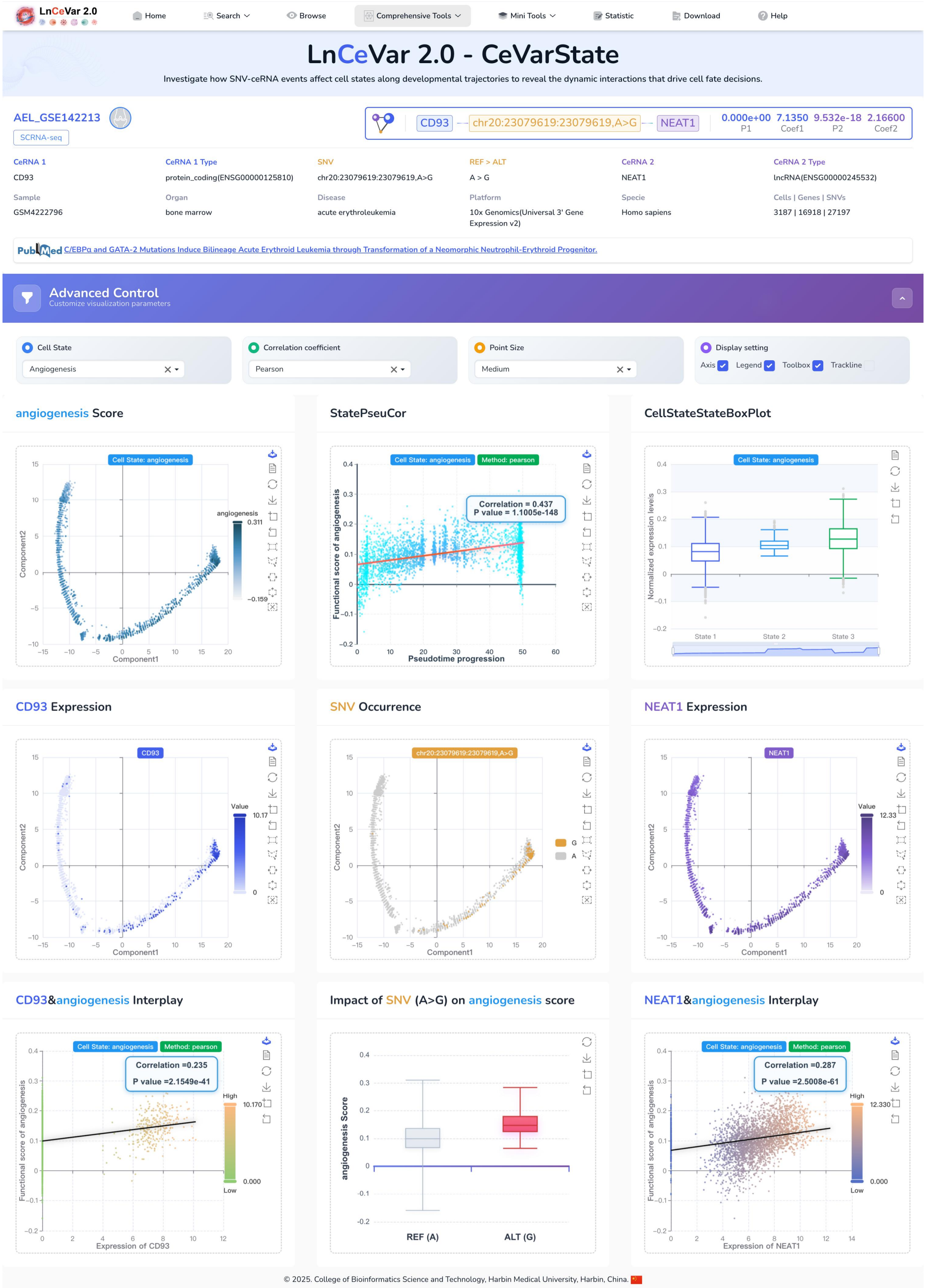

Figure S13. The *CeVarState* tool for exploring regulatory impacts of SNV-ceRNA events on cellular function heterogeneity by evaluating cell states (e.g., apoptosis, cell cycle, EMT) and their associations with SNV genotypes and ceRNA expression across functional contexts.

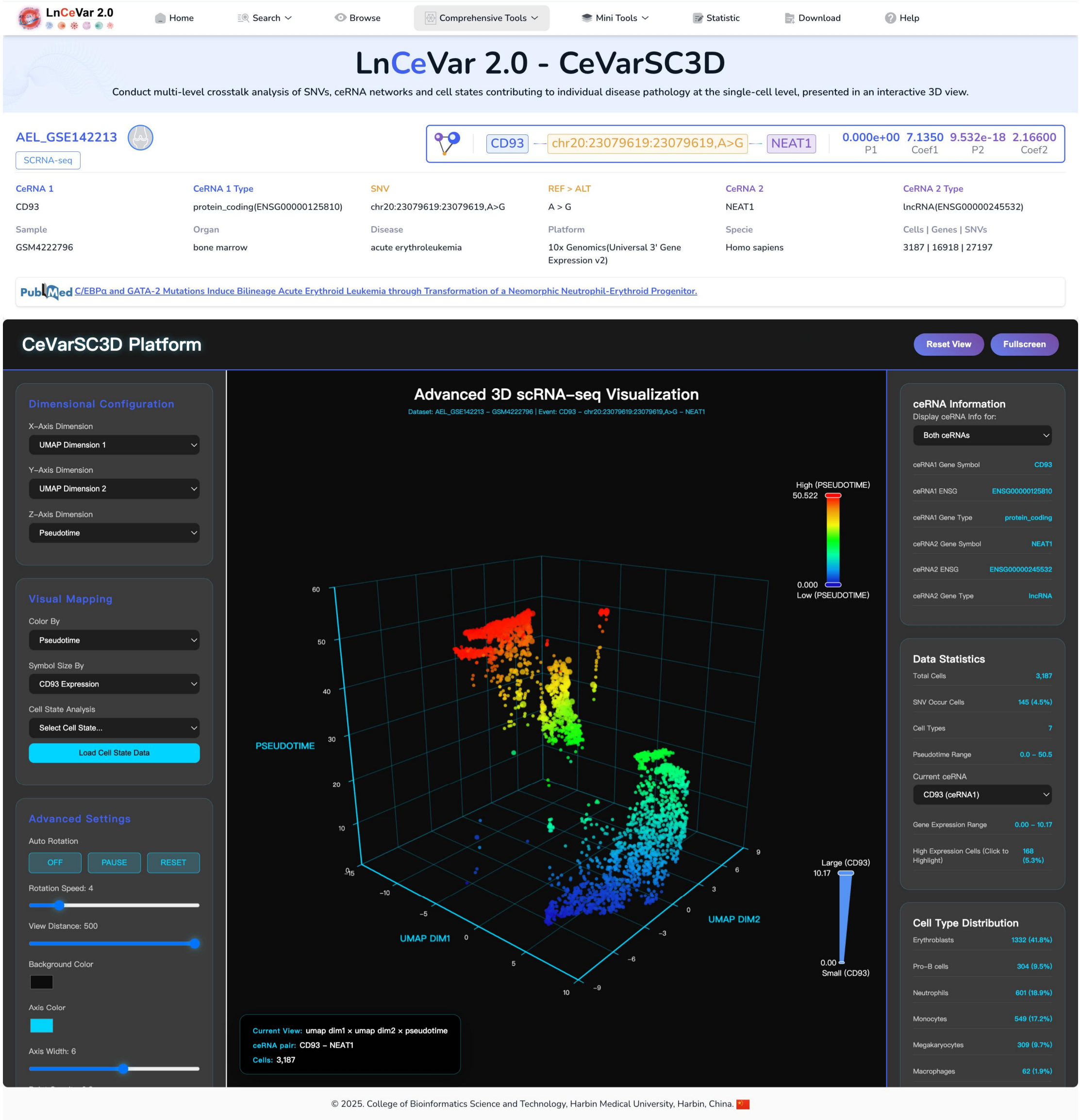

Figure S14. The *CeVarSC3D* and *CeVarST3D* tools for multi-level crosstalk analyses of SNVs, ceRNA networks, and cell states in disease pathology at single-cell and spatial levels, via interactive 3D views.
